# Association of endothelial dysfunction with incident prediabetes, type 2 diabetes and related traits: the KORA F4/FF4 study

**DOI:** 10.1136/bmjdrc-2020-001321

**Published:** 2020-07-20

**Authors:** Marie-Theres Huemer, Cornelia Huth, Florian Schederecker, Stefanie J Klug, Christa Meisinger, Wolfgang Koenig, Wolfgang Rathmann, Annette Peters, Barbara Thorand

**Affiliations:** 1 Institute of Epidemiology, Helmholtz Zentrum München, German Research Center for Environmental Health (GmbH), Neuherberg, Germany; 2 Department of Sport and Health Sciences, Chair of Epidemiology, Technical University Munich, Munich, Germany; 3 German Center for Diabetes Research, Neuherberg, Germany; 4 Independent Research Group Clinical Epidemiology, Helmholtz Zentrum München, German Research Center for Environmental Health, Neuherberg, Germany; 5 Chair of Epidemiology at UNIKA-T Augsburg, Ludwig-Maximilians-Universität München, Augsburg, Germany; 6 Institute of Epidemiology and Medical Biometry, Ulm University, Ulm, Germany; 7 Deutsches Herzzentrum München, Technische Universität München, Munich, Germany; 8 DZHK (German Centre for Cardiovascular Research), Partner Site Munich Heart Alliance, Technische Universität München, Munich, Germany; 9 Institute of Biometrics and Epidemiology, German Diabetes Center, Leibniz Center for Diabetes Research, Heinrich Heine University Düsseldorf, Düsseldorf, Germany

**Keywords:** type 2 diabetes, endothelial dysfunction, insulin resistance, glucose

## Abstract

**Introduction:**

Peripheral arterial tonometry (PAT) is an operator-independent and non-invasive measurement method to assess microvascular endothelial function in the fingertips. PAT-derived measures of endothelial function were associated with type 2 diabetes in cross-sectional studies. However, longitudinal studies are lacking. The study aims to investigate the association of two PAT-derived endothelial function parameters reactive hyperemia index (RHI) and mean baseline amplitude (MBA) with follow-up glucose and insulin parameters and the development of (pre)diabetes and type 2 diabetes.

**Research design and methods:**

The study included 673 participants initially without diabetes (328 men and 345 women) aged 52–71 years from the prospective population-based Cooperative Health Research in the Region of Augsburg F4/FF4 cohort study conducted in Southern Germany (baseline examination F4: 2006–2008; follow-up FF4: 2013–2014). An oral glucose tolerance test was performed at baseline and follow-up to define type 2 diabetes, prediabetes, fasting glucose, fasting insulin, 2-hour glucose, homeostasis model assessment of insulin resistance (HOMA-IR), homeostasis model assessment of beta-cell function and hemoglobin A1c.

**Results:**

In multivariable adjusted logistic/linear regression models, a 1 SD increase in baseline RHI was inversely associated with incident type 2 diabetes (OR 0.69 (95% CI 0.48 to 0.97)) as well as with fasting insulin (β −0.069 (95% CI −0.131 to −0.007)) and HOMA-IR (β −0.072 (95% CI −0.133 to −0.010)) at follow-up in participants with initial normoglycemia. A 1 SD increase in baseline MBA was positively associated with incident (pre)diabetes (OR 1.62 (95% CI 1.25 to 2.11)) and fasting glucose (β 0.096 (95% CI 0.047 to 0.146)) at follow-up in participants with initial normoglycemia.

**Conclusions:**

Microvascular endothelial dysfunction seems to be involved in the development of early derangements in glucose metabolism and insulin resistance and could thereby trigger the development of prediabetes and type 2 diabetes.

**Significance of this study**

**What is already known about this subject?**

- Reactive hyperemia index (RHI) and pulse amplitude, parameters of microvascular endothelial dysfunction, were associated with type 2 diabetes and related parameters in previous cross-sectional studies.

**What are the new findings?**

- Baseline measurement of RHI was inversely associated with the development of incident type 2 diabetes.
- The association was attenuated after adjusting for fasting insulin and homeostasis model assessment of insulin resistance, suggesting a potential mediating role of insulin resistance.
- Baseline measurement of pulse amplitude (MBA) was positively associated with incident (pre)diabetes and fasting glucose at follow-up in participants with initial normoglycemia.
- RHI and MBA seem to measure different microvascular endothelial mechanisms in relation to the development of diabetes.

**How might these results change the focus of research or clinical practice?**

- Besides measurements of macrovascular, also measurements of microvascular endothelial dysfunction appear to be involved in the development of incident type 2 diabetes and (pre)diabetes.

## Introduction

The endothelium plays a substantial role in regulating circulatory blood flow, fibrinolysis and coagulation within the vessels.^1–3^ Endothelial dysfunction is defined as impaired homeostasis between vasoconstriction and vasodilation of the vessels leading to decreased dilation of the vessel, inflammation and clot formation.^4 5^ As endothelial dysfunction is the first stage of atherosclerosis,^4 6^ it represents an early target to prevent cardiovascular risk factors in patients with insulin resistance or type 2 diabetes.^7^ Using measures of endothelial function, arterial abnormalities can be detected even before structural changes of the vessel wall and clinical symptoms develop. Moreover, improvements in endothelial function are linked to reduced cardiovascular morbidity and mortality.^8^ This provides a great opportunity in terms of prevention, especially because endothelial dysfunction is reversible in contrast to manifest cardiovascular diseases.^6 8^

To assess endothelial function, we used peripheral arterial tonometry (PAT), a non-invasive method, which measures the pulse volume, that is, blood flow within the index fingers before and after a 5 min cuff occlusion of the brachial artery. The resulting variables include the mean baseline amplitude (MBA) reflecting the baseline change in blood flow during pulse waves, and the reactive hyperemia index (RHI) representing the reactivity of the endothelium to the occlusion. MBA and RHI are inversely correlated.^9^ A lower MBA and a higher RHI are assumed to reflect a better microvascular endothelial function. A more frequently used alternative technique for measurement of the stimulated endothelial reactivity is the flow-mediated dilation (FMD). Both techniques are based on the same physiological measurements in terms of initiating ischemia in the arm to trigger reactive vasodilation.^10^ The substantial difference between these two methods constitutes that the PAT method measures the microvascular function, whereas FMD measures the function of large arteries.^11^ Although both measure the endothelial function, the methods should not be used interchangeably because, as they measure endothelial function parameters in different vascular beds, PAT and FMD relate to different cardiovascular risk factors. One of the reasons for using the PAT method within this project is the advantage of operator independence in contrast to FMD.^6 12 13^ Therefore, PAT is easier to implement within large population-based studies. A further advantage of the PAT is that the contralateral arm serves as an internal control to increase the validity of the RHI measurements.^10^

Endothelial dysfunction is contemplated to be an important factor in the pathogenesis of type 2 diabetes, possibly linked through hyperglycemia,^7^ insulin resistance,^13^ beta-cell dysfunction^14^ and mitochondrial DNA damage,^15^ which increase oxidative stress, inflammation and luminal obstruction of the large arteries. However, previous cross-sectional studies yielded contradictory results in linking PAT-measured microvascular endothelial dysfunction to type 2 diabetes as well as related glucose and insulin parameters.^9 11 16–19^ The prospective Monitoring of Trends and Determinants in Cardiovascular Disease/Cooperative Health Research in the Region of Augsburg (KORA) S1-S3 case-cohort study revealed an association between endothelial dysfunction and risk of type 2 diabetes using the biomarkers soluble E-selectin and soluble intracellular adhesion molecule-1, which are known to be associated with endothelial dysfunction.^20^ Other prospective studies, using biomarkers of endothelial dysfunction, support these results,^21 22^ and endothelial dysfunction ranked among the top five most important pathways in a recent biomarker-based analysis of the relevance of 19 etiological pathways.^23^ Longitudinal studies, which use FMD or more direct measures of endothelial function, such as acetylcholine-stimulated forearm blood flow, are scarce.^24 25^ A prospective cohort study investigating postmenopausal women demonstrated a significant increase in the relative risk of type 2 diabetes with decreased FMD.^24^ In a further prospective study with hypertensive patients, the maximal vasodilatory response to intra-arterial infusion of acetylcholine was inversely associated with incident type 2 diabetes independent of traditional and emerging cardiovascular risk factors.^25^ We are not aware of any study, which investigated the association between microvascular endothelial function and incident type 2 diabetes prospectively in a population-based sample using the PAT method. Therefore, the present study aimed to examine the longitudinal association of the two endothelial function parameters RHI and MBA with the development of type 2 diabetes and (pre)diabetes, as well as parameters of glucose metabolism and insulin resistance over a follow-up period of 7 years.

## Methods

### Study population

**Figure 1:**
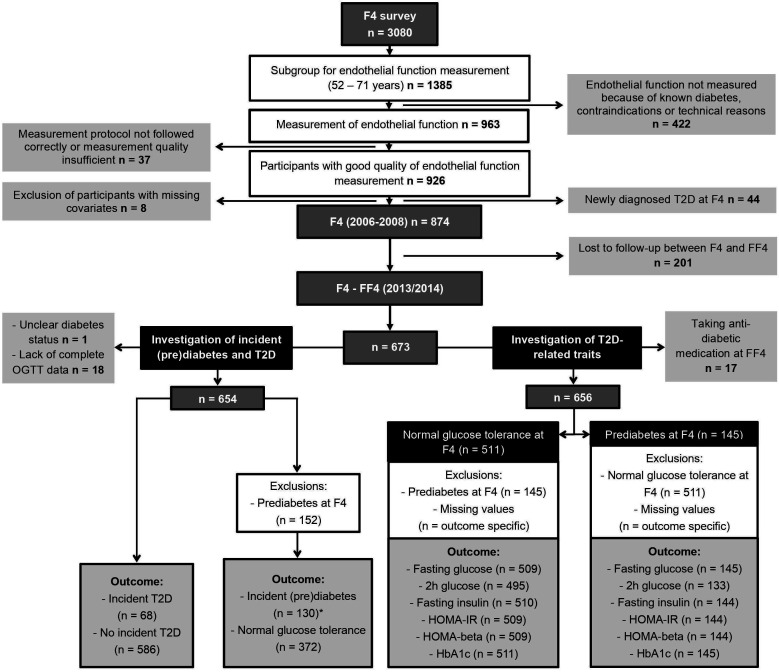
Flow chart of study participants and exclusions. *Including 111 participants with prediabetes at FF4 and 19 participants with type 2 diabetes at FF4. HbA1c, hemoglobin A1c; HOMA-beta, homeostasis model assessment of beta-cell function; HOMA-IR, homeostasis model assessment of insulin resistance; OGTT, oral glucose tolerance test; T2D, type 2 diabetes.

Data were derived from the population-based KORA F4/FF4 study, conducted in Southern Germany. For the baseline study KORA S4 (1999–2001), 6640 persons were selected out of the target population of all German citizens, aged 25–74 years living in the region of Augsburg, based on population registries of the city of Augsburg and two adjacent counties. Out of these, 4261 individuals participated in the S4 baseline examinations. The KORA F4 study (2006–2008) is the first follow-up of the KORA S4 baseline study and included 3080 participants (1486 men and 1594 women) aged 32–81 years. The second follow-up study FF4 (2013/14) included 2161 participants.^26^ For the present analysis, we used data from F4 as the baseline (exposure, covariates) and from FF4 as the follow-up (outcome). PAT measurements at F4 were only performed in the subgroup of participants aged 52–71 years, without clinically diagnosed diabetes or any contraindications (eg, hemophilia and thrombosis) (figure 1). All participants lost to follow-up were excluded from all analysis. All missing data were excluded for the respective analysis. Details concerning participant exclusions are illustrated in figure 1.

### Exposure

All participants received physical and medical examinations at the F4 and FF4 surveys by trained medical staff.

Endothelial function was assessed using PAT with the Endo-PAT2000 device produced by the company Itamar Medical (Caesarea, Israel). The system is computer-based, non-invasive and it calculates the test results automatically, which makes the measurement operator-independent. The Endo-PAT2000 uses a finger plethysmograph consisting of sensors, which measure the dynamics and extent of tone changes at the tips of both index fingers through the amplitude of the pulse waves. A pulse wave amplitude is the ‘difference between the highest and lowest point of a pulse wave’.^27^ The pulse wave amplitude was measured 5 min before, during and 5 min after a 5 min occlusion of the brachial artery of the non-dominant upper arm. The values for the premeasurements and postmeasurements were calculated separately out of the mean of the pulse wave amplitudes during the 5 min preocclusion and postocclusion. The non-occluded arm served as the control. The RHI was calculated as ((pulse amplitude of the occluded arm postocclusion/pulse amplitude of the occluded arm preocclusion)/(pulse amplitude of the control arm postocclusion/pulse amplitude of the control arm preocclusion))×baseline correction. The MBA was calculated as the mean of the pulse wave amplitudes of the occluded and control arms preocclusion. The PAT measurements were performed after the participants had received the oral glucose tolerance test (OGTT) and eaten a standardized breakfast. A detailed description of the PAT measurements is given in the online supplementary file.

10.1136/bmjdrc-2020-001321.supp1 Supplementary data

### Outcomes

Known diabetes was defined based on the participant’s self-report and subsequent validation by the responsible physician or medical chart review, or self-reported current use of glucose-lowering medication. All participants without known diabetes received the OGTT.^28^ The OGTT was performed after an overnight fast of at least 8 hours. During the fast only drinking water was allowed.^29^ The participants received 75 g carbohydrates as a 300 mL sugar solution. They ingested the solution in the morning, not later than 11:00 a.m. and drank the solution within 5 min. The participants received a blood withdrawal before and 2 hours after the carbohydrate intake. The blood samples were centrifuged and kept cool at 4°C until analysis in the study center, which was maximally 6 hours after the samples were taken.^29^ The categorical parameters newly diagnosed type 2 diabetes, prediabetes (including isolated impaired fasting glycemia (i-IFG), isolated impaired glucose tolerance (i-IGT) and the combination of IFG/IGT) and normal glucose tolerance (NGT) were defined according to 1999 WHO diagnostic criteria^30^ (detailed description in the online supplementary file). The binary outcome incident type 2 diabetes included newly diagnosed OGTT-based diabetes and known type 2 diabetes (diagnosed between F4 and FF4) identified at FF4. The corresponding participants without incident type 2 diabetes comprised participants with NGT, i-IFG, i-IGT and IFG/IGT at FF4 follow-up. The outcome incident (pre)diabetes included newly diagnosed OGTT-based type 2 diabetes, known type 2 diabetes, i-IFG, i-IGT and IFG/IGT. Participants with NGT at F4 and FF4 represented the reference group.

The continuous parameters fasting glucose, fasting insulin and hemoglobin A1c (HbA1c) were assessed in fasting blood samples obtained before the OGTT. HbA1c was measured using ion-exchange high-performance liquid chromatography in whole blood using the Adams HA 8160 Hemoglobin Analysis System (Arkray, distributed by A. Menarini Diagnostics, Florence, Italy) in KORA F4 and the Variant II Turbo HbA1c Kit-2.0 (Bio-Rad Laboratories, Hercules, USA) in KORA FF4. In KORA F4, glucose was measured using the hexokinase method (GLU Flex, Dade Behring, Deerfield, Illinois, USA). In KORA FF4, glucose was measured by an enzymatic, colorimetric method using the GLU assay on a Dimension Vista 1500 instrument (Siemens Healthcare Diagnostics, Newark, USA) or GLUC3 assay, on a Cobas c702 instrument (Roche). In KORA F4, insulin was determined in thawed serum by an electrochemiluminescence immunoassay on a Cobas e602 instrument (Roche Diagnostics, Mannheim, Germany). In KORA FF4, insulin was measured with solid-phase enzyme-labeled chemiluminescent immunometric assay on an Immulite 2000 systems analyzer (Siemens) or with electrochemiluminescence immunoassay on a Cobas e602. The instruments that were used to determine insulin and glucose changed in KORA FF4 from Siemens to Roche halfway during the study. No calibration was needed for glucose. The Siemens insulin data were calibrated using a formula developed from 194 FF4 samples, which were measured with both instruments.^28^

Homeostasis model assessment of insulin resistance (HOMA-IR) was calculated using the formula: (fasting insulin (mU/L)×fasting glucose (mmol/L))/22.5 and homeostasis model assessment of beta-cell function (HOMA-beta) using the formula: (fasting insulin (mU/L)×20)/(fasting glucose (mmol/l)–3.5).

### Covariates

Trained medical staff assessed information on sociodemographic data (eg, age, sex and education), lifestyle (eg, smoking, physical activity and alcohol intake), medication use, disease history and parental history of diabetes during a standardized face-to-face interview in the F4 survey. Detailed information describing the measurements and variable classifications of the covariates can be found in the online supplementary file.

### Statistical analysis

All statistical analyses were performed using R, V.3.4.4 (R Core Team. R: a language and environment for statistical computing. R Foundation for Statistical Computing, Vienna, Austria, 2018). RHI and MBA were standardized by dividing the values by their respective SD. The outcomes fasting insulin, HOMA-IR and HOMA-beta, as well as the covariates triglycerides and high-sensitivity C reactive protein (hsCRP), were transformed using natural logarithmic transformation. All continuous outcome variables (fasting glucose, fasting insulin, 2-hour glucose, HOMA-IR, HOMA-beta and HbA1c) were z-standardized by subtracting the mean from the values and dividing the difference by the respective SD. The associations of RHI and MBA with incident (pre)diabetes and incident type 2 diabetes were calculated using multivariable logistic regression models. For the associations of RHI and MBA with the continuous glucose and insulin parameters, we used multivariable linear regression models. Interactions between RHI or MBA and glucose tolerance status at baseline were assessed in the full study sample by inclusion of interaction terms in the linear regression models. Due to observed interactions in some models, all analyses regarding the continuous outcomes were stratified according to glucose tolerance status at baseline (ie, NGT and prediabetes). All regressions were adjusted using possible confounders based on literature research in two consecutive models. Model 1 included sex, age and the baseline value (at F4) of the continuous outcomes fasting glucose, fasting insulin, 2-hour glucose, HOMA-IR, HOMA-beta and HbA1c (only for linear regressions). Model 2 comprised waist circumference, height, triglycerides, total cholesterol/high-density lipoprotein (all continuous), actual hypertension (two categories), smoking status (three categories), alcohol intake (three categories), physical activity (four categories), low education (two categories), hsCRP (continuous) and parental history of diabetes (three categories). The logistic regression model assessing the association of RHI with incident type 2 diabetes (model 2) was further adjusted for baseline HOMA-IR and fasting insulin (in two separate models) to investigate their role as potential mediators of the association. The logistic regression model assessing the association of MBA with (pre)diabetes (model 2) was further adjusted for baseline fasting glucose. Test results with two-sided p value <0.05 were considered statistically significant.

## Results

Table 1 summarizes the characteristics of the participants of the whole study population (n=673) and stratified by the two main outcomes of our study, that is, incident type 2 diabetes versus no incident type 2 diabetes and incident (pre)diabetes versus NGT. RHI was lower and MBA higher in participants with incident type 2 diabetes compared with participants without incident type 2 diabetes as well as in participants with incident (pre)diabetes compared with participants with NGT. In partial correlation analysis, MBA and RHI were inversely correlated after adjustment for age and sex (estimate: −0.412, p=7.87e-29).

**Table 1:** Baseline characteristics of the analyzed population

Characteristics	Total n=673	Incident type 2 diabetes			Incident (pre)diabetes		
		No n=586	Yes n=68	P value*	No n=372	Yes n=130	P value*
Variables measured at baseline (F4)							
Age (years)†	60.6±5.6	60.3±5.5	63.1±5.3	<0.001	59.9±5.6	61.0±5.2	0.030
Sex male, n (%)	328 (48.7)	282 (48.1)	41 (60.3)	0.076	154 (41.4)	78 (60.0)	<0.001
Height (cm)†	168.1±8.9	168.2±8.9	168.3±8.8	0.778	167.8±8.9	168.3±8.4	0.339
Waist circumference (cm)†	94.2±13.3	93.5±13.3	102.1±11.2	<0.001	90.6±11.7	97.7±11.1	<0.001
Triglycerides (mmol/L)‡	1.26 (1.02)	1.23 (1.05)	1.63 (1.15)	<0.001	1.13 (1.07)	1.35 (1.12)	0.001
Total cholesterol/HDL†	4.15±1.1	4.11±1.12	4.70±1.17	<0.001	3.92±1.08	4.44±1.14	<0.001
Hypertension, n (%)	266 (39.5)	221 (37.7)	40 (58.8)	0.001	114 (30.6)	63 (48.5)	<0.001
Smoking status, n (%)				0.103			0.536
Never	295 (43.8)	248 (42.3)	38 (55.9)		156 (41.9)	60 (46.2)	
Former	287 (42.7)	258 (44)	23 (33.8)		164 (44.1)	50 (38.5)	
Current	91 (13.5)	80 (13.7)	7 (10.3)		52 (14.0)	20 (15.4)	
Alcohol intake, n (%)				0.976			0.798
0 g/day	185 (27.5)	159 (27.1)	19 (27.9)		103 (27.7)	40 (30.8)	
Men 0.1–39.9 g/day Women 0.1–19.9 g/day	344 (51.1)	300 (51.2)	35 (51.5)		192 (51.6)	64 (49.2)	
Men ≥40 g/day Women ≥20 g/day	144 (21.4)	127 (21.7)	14 (20.6)		77 (20.7)	26 (20.0)	
Physical activity, n (%)				0.114			0.201
No activity	170 (25.3)	143 (24.4)	21 (30.9)		83 (22.3)	32 (24.6)	
Low activity	78 (11.6)	65 (11.1)	10 (14.7)		35 (9.4)	19 (14.6)	
Moderate activity	234 (34.8)	203 (34.6)	26 (38.2)		133 (35.8)	47 (36.2)	
High activity	191 (28.4)	175 (29.9)	11 (16.2)		121 (32.5)	32 (24.6)	
Education ≤10 years, n (%)	319 (47.4)	270 (46.1)	41 (60.3)	0.036	166 (44.6)	67 (51.5)	0.208
High-sensitivity C reactive protein (mg/L)‡	1.20 (1.04)	1.16 (1.10)	1.77 (1.32)	0.002	0.99 (1.12)	1.53 (1.23)	<0.001
Parental history of diabetes, n (%)				0.104			0.003
No parent	368 (54.7)	329 (56.1)	28 (41.2)		228 (61.3)	56 (43.1)	
Unknown	134 (19.9)	113 (19.3)	16 (23.5)		60 (16.1)	36 (27.7)	
One parent	155 (23.0)	131 (22.4)	21 (30.9)		76 (20.4)	34 (26.2)	
Both parents	16 (2.4)	13 (2.2)	3 (4.4)		8 (2.2)	4 (3.1)	
MBA†	776±467	758±459	959±483	<0.001	647±426	949±468	<0.001
RHI†	2.13±0.67	2.17±0.68	1.89±0.55	<0.001	2.23±0.70	2.05±0.63	0.005
Fasting glucose (mmol/L)†	5.3±0.52	5.3±0.47	5.9±0.55	<0.001	5.1±0.39	5.4±0.37	<0.001
Fasting insulin (µU/mL)‡	9.2 (1.02)	8.9 (1.02)	13.1 (1.07)	<0.001	8.0 (1.02)	10.3 (1.04)	<0.001
2-hour glucose (mmol/L)†	6.2±1.69	6.0±1.54	8.1±1.77	<0.001	5.4±1.13	6.3±1.0	<0.001
HOMA-IR‡	2.16 (1.02)	2.06 (1.02)	3.41 (1.07)	<0.001	1.82 (1.03)	2.46 (1.04)	<0.001
HOMA-beta‡	105 (1.02)	104 (1.02)	114 (1.07)	0.185	102 (1.02)	110 (1.04)	0.102
HbA1c (mmol/mol)†	36.5±3.53	36.0±3.34	39.6±3.38	<0.001	35.4±3.19	37.2±3.13	<0.001
HbA1c (%)†	5.5±0.32	5.4±0.29	5.8±0.32	<0.001	5.4±0.28	5.5±0.28	<0.001
Variables measured at follow-up (FF4)							
Fasting glucose (mmol/L)†	5.7±0.81§	5.5±0.54	6.8±1.52	<0.001	5.3±0.40	6.0±0.58	<0.001
Fasting insulin (µU/mL)‡	10.3 (1.02)§	9.9 (1.05)¶	14.9 (1.17)	<0.001	8.7 (1.05)	13.4 (1.12)	<0.001
2-hour glucose (mmol/L)†	6.7±2.41**	6.3±1.73	12.3±3.44††	<0.001	5.4±1.09	8.5±1.87‡‡	<0.001
HOMA-IR‡	2.53 (1.02)§§	2.41 (1.05)¶	4.54 (1.20)¶¶	<0.001	2.01 (1.07)	3.54 (1.12)***	<0.001
HOMA-beta‡	101 (1.02)§§	102 (1.05)¶	99 (1.20)¶¶	0.862	100 (1.05)	109 (1.12)***	0.117
HbA1c (mmol/mol)†	37.1±4.81†††	36.4±3.67	42.8±8.2‡‡‡	<0.001	35.9±3.16	37.9±4.22	<0.001
HbA1c (%)†	5.6±0.44†††	5.5±0.34	6.1±0.75‡‡‡	<0.001	5.4±0.29	5.6±0.39	<0.001

*For differences between groups: Kruskal-Wallis test for continuous variables; χ² test for categorical variables.

†Continuous variables are presented as arithmetic mean±SD.

‡Natural logarithmic transformed variables are presented as geometric mean (antilog of SE).

§n=671.

¶n=585.

**n=628.

††n=42.

‡‡n=123.

§§n=653.

¶¶n=54.

***n=127.

†††n=672.

‡‡‡n=67.

HbA1c, hemoglobin A1c; HDL, high-density lipoprotein; HOMA-beta, homoeostasis model assessment of beta-cell function; HOMA-IR, homoeostasis model assessment of insulin resistance; MBA, mean baseline amplitude; RHI, reactive hyperemia index.

**Figure 2:**
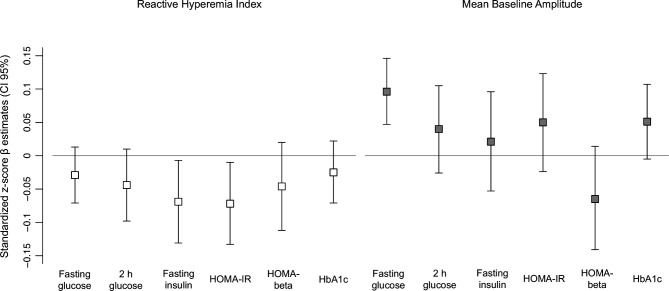
Association of baseline RHI and MBA with follow-up insulin and glucose parameters. Standardized z-score β estimates with 95% CIs for the association of insulin and glucose parameters per 1 SD increase in RHI and MBA estimated by multivariable linear regression models (only participants with NGT at baseline). Results are from model 2, adjusted for age, sex, the baseline value (at F4) of the outcome variables, waist circumference, height, triglycerides, total cholesterol/high-density lipoprotein, hypertension, smoking status, alcohol intake, physical activity, years of education, high-sensitivity C reactive protein and parental history of diabetes. HbA1c, hemoglobin A1c; HOMA-beta, homeostasis model assessment of beta-cell function; HOMA-IR, homeostasis model assessment of insulin resistance; MBA, mean baseline amplitude; NGT, normal glucose tolerance; RHI, reactive hyperemia index.

Baseline RHI was inversely associated with incident type 2 diabetes at follow-up in model 2 (OR 0.69 (95% CI 0.48 to 0.97)) (table 2). The association was attenuated after further adjustment for baseline HOMA-IR (OR 0.73 (95% CI 0.49 to 1.03)) or insulin (OR 0.71 (95% CI 0.48 to 1.01)). In participants with NGT at baseline, baseline RHI was significantly inversely associated with fasting insulin (β −0.069 (95% CI −0.131 to −0.007)) and with HOMA-IR (β −0.072 (95% CI −0.133 to −0.010)) in model 2. For fasting glucose, 2-hour glucose, HOMA-beta and HbA1c the associations were not statistically significant, but effect estimates were aligned into the same direction (figure 2, online supplementary table S1). Among participants with prediabetes at baseline, we did not observe any significant associations between RHI and the continuous outcomes (online supplementary figure S1, table S2).

**Table 2:** Association of baseline RHI and MBA with incident (pre)diabetes and incident type 2 diabetes

Model 1		Model 2	
OR (95% CI)	P value	OR (95% CI)	P value
RHI				
Incident T2D* vs no incident T2D†	0.64 (0.45 to 0.88)	0.009	0.69 (0.48 to 0.97)	0.043
Incident (pre)diabetes‡ vs NGT§	0.79 (0.63 to 0.97)	0.032	0.80 (0.63 to 1.02)	0.076
MBA
Incident T2D* vs no incident T2D†	1.37 (1.04 to 1.80)	0.024	1.20 (0.89 to 1.61)	0.238
Incident (pre)diabetes‡ vs NGT§	1.83 (1.45 to 2.32)	<0.001	1.62 (1.25 to 2.11)	<0.001

ORs per 1 SD increased RHI or increased MBA estimated by multivariable logistic regression models.

Model 1 (basic model)=age, sex.

Model 2 (further risk factors)=model 1+waist circumference, height, triglycerides, total cholesterol/high-density lipoprotein, hypertension, smoking status, alcohol intake, physical activity, years of education, high-sensitivity C reactive protein, parental history of diabetes.

*n=68.

†n=586.

‡n=130.

§n=372.

MBA, mean baseline amplitude; NGT, normal glucose tolerance; RHI, reactive hyperemia index; T2D, type 2 diabetes.

Baseline MBA was positively associated with incident (pre)diabetes (OR 1.62 (95% CI 1.25 to 2.11)) in model 2 (table 2). After adjustment for baseline glucose, the effect estimate decreased slightly (OR 1.56 (95% CI 1.20 to 2.05). In participants with NGT, MBA was also significantly positively associated with fasting glucose, 2-hour glucose, HOMA-IR, and HbA1c in model 1. However, only results for fasting glucose remained statistically significant after further adjustment (model 2) (β 0.096 (95% CI 0.047 to 0.146) (figure 2, online supplementary table S1). Among participants with prediabetes at baseline, we did not observe any significant associations between MBA and the continuous outcomes (online supplementary figure S1, table S2).

## Discussion

The present project showed that RHI was inversely associated with incident type 2 diabetes as well as HOMA-IR and fasting insulin levels at follow-up in participants who were normoglycemic at baseline. On the other hand, MBA was positively associated with incident (pre)diabetes and fasting glucose levels at follow-up in participants with initial normoglycemia. To the best of our knowledge, this is the first population-based study that assessed longitudinal associations of RHI and MBA with the development of prediabetes as well as type 2 diabetes and related traits. Our longitudinal data extend previous cross-sectional study results and suggest that microvascular endothelial dysfunction might play a role in the development of prediabetes and type 2 diabetes.

A few preceding cross-sectional studies observed significant associations of PAT-derived measures of endothelial function with type 2 diabetes.^9 11 16–19^ In interpreting the results of these studies, one has to take into account that some of them used somewhat divergent parameters of endothelial function including the F-RHI, a natural logarithmic transformation of the RHI without baseline correction. Also with regard to the MBA, slightly different parameters have been used. For instance, some studies used the baseline pulse amplitude, representing the MBA of only the occluded arm (specified only in some studies). However, as the F-RHI and the RHI as well as the baseline pulse amplitude and the MBA are highly correlated with each other, their results are assumed to be comparable. Generally in line with our longitudinal study, two cross-sectional studies displayed that baseline pulse amplitude was positively and F-RHI was inversely associated with diabetes in age-adjusted and sex-adjusted models.^9 11^ Another study investigating a Brazilian population found a positive association of baseline pulse amplitude with diabetes in age-adjusted and sex-adjusted models.^16^ While in the same study the inverse association of F-RHI with diabetes was not statistically significant in the age-adjusted and sex-adjusted model, F-RHI was significantly inversely associated with fasting glucose, further supporting a link between endothelial function and glucose metabolism.^16^ In contrast, another study found no association of diabetes with F-RHI, RHI and baseline pulse amplitude in age-adjusted and sex-adjusted models.^18^ However, this study was based on a Japanese population and the participants were at high risk for cardiovascular events. Another study displayed that self-reported history of diabetes was not associated with RHI and F-RHI after adjustment for age, sex and further nine diabetes-related traits.^19^ Furthermore, prior studies investigated the cross-sectional association of continuous insulin and glucose parameters with endothelial function parameters.^9 11 16–19^ Baseline pulse amplitude was positively and F-RHI was inversely associated with fasting glucose in age-adjusted and sex-adjusted models in two cross-sectional studies.^9 11^ Two of the three studies that found no association of endothelial function parameters with diabetes, did also not find an association with the glucose parameters.^18 19^ Causes of the conflicting results of the association of diabetes and related parameters with endothelial function parameters could be different ethnicities of the participants as well as different sample sizes and number of participants with diabetes.

The endothelial function parameter RHI is contemplated to reflect the reactive dilation of the endothelium through vasodilators such as nitric oxide (NO). This was for instance displayed in a study using healthy humans, which showed that acute inhibition of NO synthase did not influence baseline pulse amplitude (MBA) but significantly decreased reactive hyperemia assessed through PAT. Baseline pulse amplitude (MBA) changed significantly by infusion with the vasoconstrictor phenylephrine, whereas reactive hyperemia did not change. Moreover, phenylephrine acts independently of NO.^31^ This could indicate that MBA and RHI reflect different endothelium-related functions, which might relate to why MBA and RHI showed differing associations with incident (pre)diabetes and type 2 diabetes in the present study. As experimental data explaining the link between endothelial function and the continuum of dysglycemia are very limited, we can only speculate about potential mechanisms: in endothelial cells, reduced bioavailability of NO (less vasodilation, measured with RHI) contributes to insulin resistance.^32^ Moreover, the reduction of NO appears early in the development of insulin resistance and the anti-inflammatory effect of NO signaling is related to improvement of insulin resistance in multiple organs.^33^ In the present study, persons with incident prediabetes were less insulin resistant compared with persons with incident type 2 diabetes, which might explain the lacking association of RHI with incident (pre)diabetes. Of note, lower RHI values were significantly associated with incident type 2 diabetes and with higher fasting insulin and HOMA-IR levels at follow-up in participants with initial normoglycemia. Furthermore, adjustments for fasting insulin as well as HOMA-IR attenuated the association between RHI and incident type 2 diabetes, suggesting a possible mediating role of insulin resistance. This further underlines the concept that RHI reflects NO reduction, which promotes insulin resistance leading over time to manifested diabetes. An intervention study supports this concept as they proposed that changes in HOMA-IR through exercise training were independently correlated with changes in RHI in patients with obesity.^34^ Along these lines, experimental data in mouse models of obesity demonstrated that reduced glucose uptake within the skeletal muscle is caused by impaired insulin signaling in endothelial cells due to reduced insulin-induced endothelial NO (vasodilator) synthase phosphorylation and insulin receptor substrate 2 expression. This leads to decreased insulin delivery to the skeletal muscle, reducing the uptake of glucose into skeletal muscle^35^ and in the long run possibly to the manifestation of type 2 diabetes. The lack of association between MBA and HOMA-IR as well as fasting insulin could potentially explain the missing link to type 2 diabetes development.

The MBA reflects the pulse waves, that is, pressure changes, which result from volume differences due to changes in the arteriolar volume of the finger.^27^ Higher pressure and volume changes could reflect increased blood flow. This is based on the observation that after the release of the blood pressure cuff the pulse amplitude increases compared with baseline values^9^ because the release of the cuff provokes increased blood flow. Without this external obstruction an increased blood flow might also be necessary in other conditions which obstruct the vessel for a longer time in order to still transport enough oxygen to demanding structures. Molecules, such as leukocytes binding to the vessel wall, decrease the space of the bloodstream and lead to obstruction.^5^ As the endothelium’s function is to regulate blood flow^13^ and blood fluidity,^1^ baseline pulse amplitude (MBA) might be an important indicator of microvessel function in addition to microvessel structure.^36^ Higher MBA values could reflect the increased blood flow and therefore severity of the vessel obstruction.

Previous studies have suggested that higher glucose levels impair endothelial function,^37 38^ possibly because elevated glucose levels increase platelet aggregation, inhibit fibrinolysis^39^ and lead to coagulation independent of insulin levels in healthy subjects.^40^ The underlying mechanisms include the transport of glucose into endothelial cells by diffusion independently of insulin. If endothelial cells in vitro are exposed to high glucose, they increase the production of fibronectin and procoagulant proteins and decrease fibrinolytic potential,^41^ all leading to higher obstruction within the vessel and therefore possibly increased MBA. The longitudinal results of the present study suggest these observations vice versa. Especially in persons with initial NGT, decreased microcirculation as reflected by increased MBA may also lead to increased glucose levels over time. Furthermore, elevated fasting glucose concentrations at baseline partly explained the strong association between MBA and incident (pre)diabetes suggesting a potential mediating role of fasting glucose. As other longitudinal and experimental studies addressing this potential mechanism are largely lacking, further research is necessary in this respect.

We further demonstrated that in participants with prediabetes at baseline, baseline RHI and MBA were not associated with follow-up values of the continuous traits. As we found multiple associations in persons with NGT at baseline, this might reflect that RHI and MBA are both mainly related to the early impairment of glucose metabolism and early stages of insulin resistance.

### Strengths and limitations

A major strength of this project constitutes the population-based longitudinal design with availability of standardized covariates enabling inclusion of a large number of confounders into the models. Furthermore, a standardized OGTT was performed to determine previously unknown diabetes and prediabetes cases. Measurements of endothelial function were performed in a highly standardized manner using PAT technology and only measurements with good quality were included in the present analyses. Limitations of this project include the relatively low number of participants, who developed incident type 2 diabetes during the follow-up period. Furthermore, as the study only included predominantly white Europeans between the age of 52 and 71 years, the results cannot be generalized to younger adults or other ethnicities. Furthermore, the PAT measurement represents only an indirect measurement of endothelial function.

## Conclusion

The present analysis is the first to present longitudinal data on the association of PAT-derived microvascular endothelial dysfunction with type 2 diabetes. We observed an inverse association of the microvascular endothelial function parameter RHI, which reflects reactive vasodilation, with the development of incident type 2 diabetes and a positive association of MBA reflecting blood flow with incident (pre)diabetes. Thus, microvascular endothelial dysfunction seems to be involved in the development of early derangements in glucose and insulin metabolism and may thereby trigger the development of prediabetes and type 2 diabetes.
