# Supplementary material for: Association of endothelial dysfunction with incident prediabetes, type 2 diabetes and related traits: the KORA F4/FF4 study
Source: BMJ Open Diabetes Res Care. 2020 Jul 20;8(1):e001321. doi: 10.1136/bmjdrc-2020-001321 (PMC7373312; doi:10.1136/bmjdrc-2020-001321)
Supplement: Supplementary data [file bmjdrc-2020-001321supp001.pdf]

Supplementary File -

Association of endothelial dysfunction with incident prediabetes, type 2 diabetes and related traits: the KORA F4/FF4 study

Authors: Marie-Theres Huemer [1,2], Cornelia Huth [1,3], Florian Schederecker [1], Stefanie J. Klug [2], Christa Meisinger [4,5], Wolfgang Koenig [6,7,8], Wolfgang Rathmann [3,9], Annette Peters [1,3], Barbara Thorand [1,3]

[1]Institute of Epidemiology, Helmholtz Zentrum München, German Research Center for Environmental Health (GmbH), Neuherberg, Germany

[2]Department of Sport and Health Sciences, Chair of Epidemiology, Technical University Munich, Munich, Germany

[3]German Center for Diabetes Research (DZD), Neuherberg, Germany

[4]Independent Research Group Clinical Epidemiology, Helmholtz Zentrum München, German Research Center for Environmental Health, Neuherberg, Germany

[5]Chair of Epidemiology at UNIKA-T Augsburg, Ludwig-Maximilians-Universität München, Augsburg, Germany

[6]Institute of Epidemiology and Medical Biometry, Ulm University, Ulm, Germany

[7]Deutsches Herzzentrum München, Technische Universität München, Munich, Germany

[8]DZHK (German Centre for Cardiovascular Research), partner site Munich Heart Alliance, Technische Universität München, Munich, Germany

[9]Institute of Biometrics and Epidemiology, German Diabetes Center, Leibniz Center for Diabetes Research, Heinrich Heine University Düsseldorf, Düsseldorf, Germany

Corresponding author:

Prof. Dr. Barbara Thorand

Email: thorand@helmholtz-muenchen.de

Table S1: Association of baseline RHI and MBA with follow-up insulin and glucose parameters (NGT at baseline)

	Model 1		Model 2	
RHI	β (95%CI)	P	β (95%CI)	P
Fasting glucose (mmol/l)[a]	-0.033 (-0.075, 0.008)	0.116	-0.029 (-0.071, 0.013)	0.181
2 h glucose (mmol/l)[b]	-0.052 (-0.107, 0.003)	0.066	-0.044 (-0.098, 0.010)	0.114
Fasting insulin (µU/ml))[c]	-0.062 (-0.125, 0.0004)	0.053	-0.069 (-0.131, -0.007)	**0.030**
HOMA-IR[a]	-0.066 (-0.129, -0.004)	**0.039**	-0.072 (-0.133, -0.010)	**0.023**
HOMA-beta (%)[a]	-0.037 (-0.101, 0.028)	0.270	-0.046 (-0.112, 0.020)	0.174
HbA1c (mmol/mol)[d]	-0.018 (-0.065, 0.029)	0.458	-0.025 (-0.072, 0.022)	0.296
MBA	β (95%CI)	P	β (95%CI)	P
Fasting glucose (mmol/l)[a]	0.118 (0.070, 0.166)	**<0.001**	0.096 (0.047, 0.146)	**<0.001**
2 h glucose (mmol/l)[b]	0.083 (0.018, 0.149)	**0.013**	0.040 (-0.026, 0.105)	0.238
Fasting insulin (µU/ml))[c]	0.067 (-0.007, 0.141)	0.077	0.021 (-0.053, 0.096)	0.573
HOMA-IR[a]	0.096 (0.022, 0.170)	**0.011**	0.050 (-0.024, 0.123)	0.188
HOMA-beta (%)[a]	-0.030 (-0.105, 0.046)	0.444	-0.065 (-0.143, 0.014)	0.106
HbA1c (mmol/mol)[d]	0.069 (0.014, 0.124)	**0.015**	0.051 (-0.005, 0.107)	0.078

Huemer M-T, *et al. BMJ Open Diab Res Care* 2020; 8:e001321. doi: 10.1136/bmjdrc-2020-001321

Effect estimates per one standard deviation increased RHI or increased MBA estimated by multivariable linear regression models (only participants with normal glucose tolerance at baseline).

Abbreviations: HbA1c, hemoglobin A1c; HOMA-beta, homeostasis model assessment of beta-cell function; HOMA-IR, homeostasis model assessment of insulin resistance; MBA, mean baseline amplitude; RHI, reactive hyperemia index.

Significant values are presented in bold.

Model 1 (Basic model) = age, sex, baseline value of the outcome.

Model 2 (Further risk factors) = model 1 + waist circumference, height, triglycerides, total cholesterol/HDL, hypertension, smoking status, alcohol intake, physical activity, years of education, high-sensitive C-reactive protein, parental history of diabetes.

[a] n = 509; [b] n = 495; [c] n = 510; [d] n = 511

Table S2: Association of baseline RHI and MBA with follow-up insulin and glucose parameters (prediabetes at baseline)

	Model 1		Model 2	
RHI	β (95%CI)	P	β (95%CI)	P
Fasting glucose (mmol/l)[a]	-0.076 (-0.299, 0.146)	0.504	-0.022 (-0.261, 0.218)	0.860
2 h glucose (mmol/l)[b]	-0.092 (-0.320, 0.135)	0.429	-0.085 (-0.334, 0.164)	0.504
Fasting insulin (µU/ml))[c]	0.068 (-0.045, 0.181)	0.243	0.085 (-0.034, 0.204)	0.163
HOMA-IR[a]	0.058 (-0.064, 0.179)	0.354	0.076 (-0.051, 0.203)	0.241
HOMA-beta (%)[a]	0.053 (-0.078, 0.185)	0.429	0.076 (-0.066, 0.218)	0.298
HbA1c (mmol/mol)[d]	-0.150 (-0.346, 0.046)	0.137	-0.161 (-0.365, 0.042)	0.123
MBA	β (95%CI)	P	β (95%CI)	P
Fasting glucose (mmol/l)[a]	-0.133 (-0.351, 0.086)	0.237	-0.133 (-0.377, 0.110)	0.284
2 h glucose (mmol/l)[b]	0.019 (-0.211, 0.249)	0.871	0.090 (-0.171, 0.351)	0.501
Fasting insulin (µU/ml))[c]	-0.063 (-0.173, 0.048)	0.269	-0.074 (-0.198, 0.050)	0.245
HOMA-IR[a]	-0.090 (-0.209, 0.029)	0.140	-0.090 (-0.222, 0.043)	0.187
HOMA-beta (%)[a]	0.019 (-0.110, 0.147)	0.775	-0.018 (-0.164, 0.128)	0.806
HbA1c (mmol/mol)[d]	-0.081 (-0.273, 0.111)	0.408	-0.054 (-0.263, 0.154)	0.612

Effect estimates per one standard deviation increased RHI or increased MBA estimated by multivariable linear regression models (only participants with prediabetes at baseline).

Abbreviations: HbA1c, hemoglobin A1c; HOMA-beta, homeostasis model assessment of beta-cell function; HOMA-IR, homeostasis model assessment of insulin resistance; MBA, mean baseline amplitude; RHI, reactive hyperemia index.

Model 1 (Basic model) = age, sex, baseline value of the outcome.

Model 2 (Further risk factors) = model 1 + waist circumference, height, triglycerides, total cholesterol/HDL, hypertension, smoking status, alcohol intake, physical activity, years of education, high-sensitive C-reactive protein, parental history of diabetes.

[a] n = 509; [b] n = 495; [c] n = 510; [d] n = 511

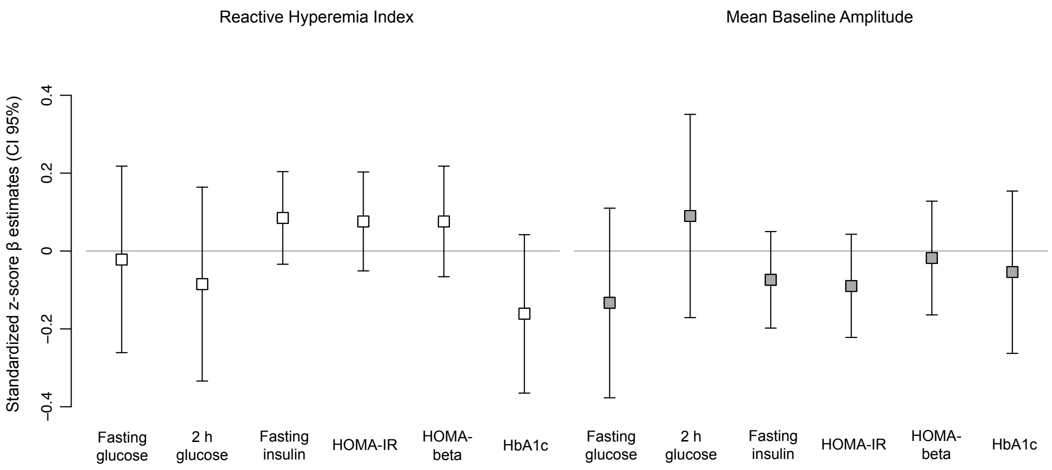

Figure S1: Association of baseline RHI and MBA with follow-up insulin and glucose parameters (prediabetes at baseline).

Standardized z-score β estimates with 95 % confidence intervals for the association of insulin and glucose parameters per one standard deviation increase in RHI and MBA estimated by multivariable linear regression models (only participants with prediabetes at baseline).

Results are from model 2 adjusted for age, sex, the baseline value (at F4) of the outcomes, waist circumference, height, triglycerides, total cholesterol/HDL, hypertension, smoking status, alcohol intake, physical activity, years of education, high-sensitive C-reactive protein, and parental history of diabetes.

Abbreviations: HbA1c, hemoglobin A1c; HOMA-beta, homeostasis model assessment of beta-cell function; HOMA-IR, homeostasis model assessment of insulin resistance; MBA, mean baseline amplitude; RHI, reactive hyperemia index.

Huemer M-T, *et al. BMJ Open Diab Res Care* 2020; 8:e001321. doi: 10.1136/bmjdrc-2020-001321

Detailed description of the exposure measurements: Peripheral arterial tonometry – EndoPAT2000

The EndoPAT2000 consists of biosensors that are attached to both index fingers creating a consistent pressure field within the upper two-thirds of the fingers. The pressure prevents distal venous blood pooling and therefore a vasoconstriction reflex. Moreover, the pressure increases the attachment of the sensors to the finger, which reduces movement artifacts. Additionally, the online display allows for real-time viewing to control if the measurement functions properly (1). The pulse wave amplitude was measured before and after occlusion of the upper arm, which was used to provoke incremental blood flow after the release of the occlusion. The principle of the measurement is based on the increase in vasodilation of the blood vessels initiated by the increased blood flow. The occlusion using a blood pressure cuff was only performed on one upper arm to occlude the brachial artery. The other arm/finger served as a control. Various studies present different positions for the fixation of the blood pressure cuff, either the forearm or the upper arm (2-4). However, there is no significant difference in the PAT ratio after occluding the forearm or the upper arm (5). Generally, the blood pressure cuff is being inflated for 5 minutes because the maximum response of blood flow occlusion is reached after 5 minutes of occlusion (5).

The exposure of interest is the endothelial function represented through the variable reactive hyperemia index (RHI), which was calculated as:

A= Mean pulse amplitude of the occluded arm post occlusion

B= Mean pulse amplitude of the occluded arm pre occlusion

C= Mean pulse amplitude of the control arm post occlusion

D= Mean pulse amplitude of the control arm pre occlusion

$$\mathrm{RHI} = \frac{\mathrm{A/B}}{\mathrm{C/D}} \times \text{Baseline Correction}$$

The value for each compartment of the calculation was based on the amplitude of the pulse waves. A mean of the amplitudes was built for every time period (baseline, post occlusion) and arm/finger (occluded, control). The A/B is the dilation index, represented by the values

pre and post occlusion of the occluded arm and the C/D is the systemic correction of the pre and post occlusion values of the control arm (no occlusion). The control arm measurements serve as a control for concurrent non-endothelial dependent changes in vascular tone. The mean pulse amplitude of the occluded arm was further used as a baseline correction to reduce the internal influence on the RHI (1).

Prior to the PAT measurement, the participants received a standardized breakfast including one bread roll or two small slices of whole-grain bread with honey or marmalade, some butter or margarine and either a cup of coffee or tea. Not allowed were sausages, cheese, and fruit juice. Before the PAT measurement started, certain exclusion criteria were assessed. Participants with a latex allergy, hemophilia, thrombosis or a shunt at the arm (dialysis patients) were not allowed to participate. The PAT measurements were performed by well-trained medical staff. The ambient conditions included a quiet and undisturbed room (no one was allowed to come in during measurements), consistent room temperature between 21 °C and 24 °C and switching off bright lighting as well as cell phones. The Endo-PAT2000 was switched on 20 minutes before the first measurement and to ensure signal transmission between sensors and laptop test samples were used. As the participant arrived, the measurement was explained and his or her hands were checked for extremely high or low temperature. If this was the case, the measurement was delayed until the participant´s hands reached normal temperature. Overall, the whole procedure lasted around 25 minutes. The participant was asked to take off jewelry, watches as well as tight clothing constricting the arm. If the participant’s fingernails were very long, he or she was asked if the operator could cut them to an appropriate length for the measurement. If this was not feasible, the lengths of the fingernails were noted within the input mask of the laptop. Before the measurement started, the participant filled out a questionnaire about recent smoking, physical activity, and nutrition. Starting the measurement, the participant laid down on an examination table on his or her back, the hand at the level of the heart. The participant was asked to stay calm and relaxed during the whole procedure. Starting the

measurement, the blood pressure cuff was fixed on the participant's non-dominant upper arm. Afterward, the sensors were attached to the index fingers as well as inflated to create the constant pressure described before. Polystyrene and mull was used to keep the fingers apart. After testing the signal quality, the baseline measurement was performed for 5 minutes. From this point on the recording started and lasted throughout the whole procedure. The blood pressure cuff was inflated at 60 mmHg over the systolic blood pressure and released after 5 minutes. The recording proceeded for another 5 minutes. Afterward, the quality of the measurement was examined, the data was saved and the participant was released.

Detailed description of the diabetes definition:

Diabetes was diagnosed if the fasting serum glucose was ≥ 7.0 mmol/l or the 2 h serum glucose was ≥ 11.1 mmol/l. An i-IGT was diagnosed if the fasting glucose was < 6.1 mmol/l and the 2 h glucose was ≥ 7.8 mmol/l but < 11.1 mmol/l. An i-IFG was determined if the fasting glucose was ≥ 6.1 mmol/l but < 7.0 mmol/l and the 2 h glucose was < 7.8 mmol/l. The combination IFG/IGT was diagnosed if fasting glucose was ≥ 6.1 mmol/l but < 7.0 mmol/l and the 2 h glucose was ≥ 7.8 mmol/l but < 11.1 mmol/l. NGT was determined if the fasting glucose was < 6.1 mmol/l and 2 h glucose < 7.8 mmol/l.

Detailed description of the covariates:

Smoking status was classified as never, former or current smoker. A current smoker was defined as smoking at least one cigarette per day. The physical activity level was estimated using two separate four-category interview questions asking about the time per week spent on sports activities during leisure time (including cycling) in summer and winter (0, <1, 1 to 2, and >2 h exercise/week). The winter and summer responses were combined to create one variable of leisure-time physical activity. The participants were categorized into either "no activity" defined as less than 1 h sports in summer and winter, "low activity" defined as irregular participation in sports for less than 1 h per week in either summer or winter and not

BMJ Publishing Group Limited (BMJ) disclaims all liability and responsibility arising from any reliance placed on this supplemental material which has been supplied by the author(s)

more than 2 h in the other season, "moderate activity" defined as regular participation in sports for about 1-2 h per week in both seasons or "high activity" defined as regular sports in summer and winter for more than 2 h per week. The variable education was defined based on the years of education, either > 10 years or ≤ 10 years. The alcohol intake was calculated in grams per day based on information regarding the intake of alcoholic beverages on the previous workday and during the previous weekend. Alcohol intake was classified into three sex-specific categories: men: 0 g/day, 0.1-39.9 g/day, and ≥ 40 g/day; women: 0 g/day, 0.1-19.9 g/day, and ≥ 20 g/day.(6) Parental history of diabetes was categorized based on self-report into either no parent, unknown, one parent or both parents. The fasting blood samples obtained before the OGTT were used to determine high-density lipoprotein (HDL), total cholesterol, triglycerides, and high-sensitive C-reactive protein (hsCRP). Total cholesterol and HDL were assessed in fresh serum by enzymatic methods (CHOL Flex and AHDL Flex, Dade Behring, Marburg, Germany).(7) Triglycerides were assessed using the Boehringer GPO-PAP assay.(8) HsCRP was measured from frozen plasma using a high-sensitive latex enhanced nephelometric assay on a BN 2 analyzer (Dade Behring, Marburg, Germany).(7) Blood pressure was measured in sitting position on the right arm three times in three-minute intervals after an at least 5-minute rest. The mean of the second and third measurement was used. Participants with a blood pressure of ≥ 140/90 mmHg or intake of antihypertensive medication given that participants were aware of having hypertension were classified as having hypertension. Weight, height, and waist circumference were measured with participants in light clothing and without shoes.(6)

Detailed description of the loss to follow up of the KORA studies S4-F4 and F4-FF4:

KORA S4: 4261 participants

- 174 deaths before KORA-F4 examination
- 6 moved to an unknown location
- 198 moved outside the study region
- 12 refused to be contacted

3871 participants eligible

- 615 refused

- 176 no contact possible

= 3080 KORA-F4 participants

KORA F4: 3080 participants

- 168 deaths before KORA-FF4 examination

- 11 moved to an unknown location

- 86 moved outside the study region

- 67 refused to be contacted

2748 participants eligible

- 539 refused

- 48 no contact possible

= 2161 KORA-FF4 participants

The response rate (including death) of all study participants from F4 to FF4 is 70.2 % (29.8 % lost to follow-up). In the present analysis, the response rate of the participants from F4 to FF4 is 75.7 % (24.3 % lost to follow-up).

Literature:

1. Itamar Medical. Assessing endothelial function overview & scientific validation of EndoPAT. 2016.
2. Brant LC, Hamburg NM, Barreto SM, Benjamin EJ, Ribeiro AL. Relations of digital vascular function, cardiovascular risk factors, and arterial stiffness: the Brazilian Longitudinal Study of Adult Health (ELSA-Brasil) cohort study. J Am Heart Assoc. 2014;3(6):e001279.

3. Hamburg NM, Keyes MJ, Larson MG, Vasan RS, Schnabel R, Pryde MM, et al. Cross-sectional relations of digital vascular function to cardiovascular risk factors in the Framingham Heart Study. Circulation. 2008;117(19):2467-74.
4. Schnabel RB, Schulz A, Wild PS, Sinning CR, Wilde S, Eleftheriadis M, et al. Noninvasive vascular function measurement in the community: cross-sectional relations and comparison of methods. Circ Cardiovasc Imaging. 2011;4(4):371-80.
5. Faizi A, Kornmo D, Agewall S. Evaluation of endothelial function using finger plethysmography. In: University AUHaO, editor. 2009.
6. Meisinger C, Thorand B, Schneider A, Stieber J, Döring A, Löwel H. Sex differences in risk factors for incident type 2 diabetes mellitus: the MONICA Augsburg cohort study. Arch Intern Med. 2002;162(1):82-9.
7. Huth C, Beuerle S, Zierer A, Heier M, Herder C, Kaiser T, et al. Biomarkers of iron metabolism are independently associated with impaired glucose metabolism and type 2 diabetes: the KORA F4 study. Eur J Epidemiol. 2015;173(5):643-53.
8. Rathmann W, Haastert B, Icks A, Lowel H, Meisinger C, Holle R, et al. High prevalence of undiagnosed diabetes mellitus in Southern Germany: target populations for efficient screening. The KORA survey 2000. Diabetologia. 2003;46(2):182-9.
